# Cancer among the Incas

**Published:** 1907-05-25

**Authors:** 


### Cancer Among the Incas.

The ancient inhabitants of South America, the Incas of Peru, are of unknown origin; but it is certain that to all intents and purposes their evolution took place upon lines quite independent of that of European man. It would be extremely interesting to know whether, previous to the invasion of the Spaniards, the general pathological processes which occurred in these Incas were similar to those of Europeans. The point is of scientific rather than of practical importance; and yet it is of some definite value to an English physician, who may practise in almost any part of the world, to know whether or not the general pathology which he studies at home is likely to hold good all the world over. It might be of considerable service, moreover, in deciding between the "microbial" and the "developmental" theories of cancer. If we could determine with certainty that malignant new growths were non-existent in South America previous to their importation from Europe, and if at the same time we could be sure that tumours of known developmental origin occurred in Incas in just the same way as they do in Europeans, it would be some argument against the developmental origin of the former. If, on the other hand, it could be definitely shown that the Incas had malignant new growths precisely similar to those of Europe, one would either have to allow that the cancer "microbes" arose by evolution in South America simultaneously with their evolution in Europe, or else, what would then seem more likely, that these neoplasms are developmental. The argument would be circumstantial rather than direct, but it would at least have great interest and value.

The first step in this direction was taken by Professor Virchow in 1886, when he wrote a paper upon "Pathological Bone-changes in the Ancient Inhabitants of Peru." In this paper he describes a collection of bones obtained from the dry, sandy burying-places of the Peruvian "Indians," near the Inca Temple of Pachacamac, some distance from the modern Lima. Amongst these bones were several that bore exostoses close to the epiphysial lines. Professor Virchow was able to prove that a femur, a tibia, a humerus, and a fibula, each of which bore spongy exostoses of various sizes and shapes, all belonged to the same individual. The position of the exostoses and their appearance were precisely the same as those seen nowadays in Europeans; in the Inca, as in the European, their origin was obviously developmental in relation to the epiphysial cartilages. This existence of multiple exostoses due to developmental abnormalities in Incas would seem to indicate that these ancient Peruvians—entirely separated as they were from other continents—were liable to precisely the same developmental tumours as those we see to-day; what we should like to know is whether they were also subject to carcinoma and sarcoma. The question is impossible of solution as regards carcinoma, for soft parts have all disappeared and only bony tissues remain; sarcoma, however, might still be found in connection with an Inca's bones—the remains of an osteo-sarcoma of the tibia, for example, could scarcely be mistaken for anything else. Few of us will ever have an opportunity of searching for the remains of a sarcoma upon an Inca skeleton, but it is just possible that somebody who reads these lines might be able to do so. The discovery of such an Inca sarcoma would, we think, go a considerable way towards proving that sarcoma, at any rate, is of developmental, and not microbial, origin.

